# Automatic Processing of Changes in Facial Emotions in Dysphoria: A Magnetoencephalography Study

**DOI:** 10.3389/fnhum.2018.00186

**Published:** 2018-05-04

**Authors:** Qianru Xu, Elisa M. Ruohonen, Chaoxiong Ye, Xueqiao Li, Kairi Kreegipuu, Gabor Stefanics, Wenbo Luo, Piia Astikainen

**Affiliations:** ^1^Research Center of Brain and Cognitive Neuroscience, Liaoning Normal University, Dalian, China; ^2^Jyväskylä Centre for Interdisciplinary Brain Research, Department of Psychology, University of Jyväskylä, Jyväskylä, Finland; ^3^Institute of Psychology, University of Tartu, Tartu, Estonia; ^4^Translational Neuromodeling Unit, Institute for Biomedical Engineering, University of Zurich–ETH Zurich, Zurich, Switzerland; ^5^Laboratory for Social and Neural Systems Research, Department of Economics, University of Zurich, Zurich, Switzerland; ^6^Laboratory of Emotion and Mental Health, Chongqing University of Arts and Sciences, Chongqing, China

**Keywords:** automatic, change detection, dysphoria, emotional faces, magnetoencephalography

## Abstract

It is not known to what extent the automatic encoding and change detection of peripherally presented facial emotion is altered in dysphoria. The negative bias in automatic face processing in particular has rarely been studied. We used magnetoencephalography (MEG) to record automatic brain responses to happy and sad faces in dysphoric (Beck’s Depression Inventory ≥ 13) and control participants. Stimuli were presented in a passive oddball condition, which allowed potential negative bias in dysphoria at different stages of face processing (M100, M170, and M300) and alterations of change detection (visual mismatch negativity, vMMN) to be investigated. The magnetic counterpart of the vMMN was elicited at all stages of face processing, indexing automatic deviance detection in facial emotions. The M170 amplitude was modulated by emotion, response amplitudes being larger for sad faces than happy faces. Group differences were found for the M300, and they were indexed by two different interaction effects. At the left occipital region of interest, the dysphoric group had larger amplitudes for sad than happy deviant faces, reflecting negative bias in deviance detection, which was not found in the control group. On the other hand, the dysphoric group showed no vMMN to changes in facial emotions, while the vMMN was observed in the control group at the right occipital region of interest. Our results indicate that there is a negative bias in automatic visual deviance detection, but also a general change detection deficit in dysphoria.

## Introduction

Depression is a common and easily recurring disorder. Decades ago, Beck (1976) suggested that negatively biased information processing plays a role in the development and maintenance of depression. According to his theory, a dysphoric mood is maintained through attention and memory functions biased toward negative information, and these cognitive biases also expose individuals to recurrent depression (Beck, 1967, 1976).

Previous empirical studies have indeed demonstrated a negative bias in attention and memory functions in depression (Ridout et al., 2003, 2009; Linden et al., 2011; for reviews see, Mathews and Macleod, 2005; Browning et al., 2010; Delle-Vigne et al., 2014). Depressed participants have a pronounced bias toward negative stimuli as well as toward sad faces (Gotlib et al., 2004; Dai and Feng, 2012; Bistricky et al., 2014).

Brain responses, such as electroencephalography (EEG) and magnetoencephalography (MEG) responses, allow face processing to be studied in a temporally accurate manner. Previous studies have demonstrated that different evoked EEG/MEG responses reflect different stages of face perception (Bourke et al., 2010; Luo et al., 2010; Delle-Vigne et al., 2014). P1 (or P100) in event-related potentials (ERPs) and its magnetic counterpart M100 are thought to reflect the encoding of low-level stimulus features and are also modulated by emotional expressions (e.g., Batty and Taylor, 2003; Susac et al., 2010; Dai and Feng, 2012). P1 is also affected by depression: sad faces elicited greater responses than neutral and happy faces in the depressed group reflecting an attentive negative bias in depression (Dai and Feng, 2012). The following N170 component in ERPs and the magnetic M170 both index the structural encoding of faces (Bentin et al., 1996; Liu et al., 2002). This component has also shown emotional modulation in some studies (for positive results, see e.g., Batty and Taylor, 2003; Miyoshi et al., 2004; Japee et al., 2009; Wronka and Walentowska, 2011; and for negative results, see, e.g., Eimer and Holmes, 2002; Herrmann et al., 2002; Eimer et al., 2003; Holmes et al., 2003). In addition, depression alters the N170/M170: some ERP studies have found a smaller N170 response in depressed participants than in healthy controls (Dai and Feng, 2012), while others have found no such effects (Maurage et al., 2008; Foti et al., 2010; Jaworska et al., 2012). Negative bias has been reported as a higher N170 amplitude for sad faces relative to happy and neutral faces in depressed participants (Chen et al., 2014; Zhao et al., 2015). The P2 component (also labeled as P250), a positive polarity ERP response approximately at 200–320 ms in the temporo-occipital region, is followed by the N170 and reflects the encoding of emotional information (Zhao and Li, 2006; Stefanics et al., 2012; Da Silva et al., 2016). It has also a counterpart in MEG responses, sometimes labeled M220 (e.g., Itier et al., 2006; Schweinberger et al., 2007; Bayle and Taylor, 2010). In an ERP study with an emotional face intensity judgment task, depressed participants showed larger P2 amplitude for sad faces than happy and neutral ones, reflecting a negative bias in their attentive level, which was not found in the control group (Dai and Feng, 2012).

Although the negative bias in depression is well documented in settings involving sustained attention (for reviews see, Mathews and Macleod, 2005; Browning et al., 2010; Delle-Vigne et al., 2014), few studies have focused on automatic processing of emotional stimuli in depressed participants. Since our adaptive behavior relies largely on preattentive cognition (Näätänen et al., 2010), it is important to investigate emotional face processing in preattentive levels in healthy and dysphoric participants.

Studies based on the electrophysiological brain response called visual mismatch negativity (vMMN), a visual counterpart of the auditory MMN (Näätänen et al., 1978), have demonstrated that automatic change detection is altered in depression (Chang et al., 2010; Qiu et al., 2011; for a review, see Kremláček et al., 2016). vMMN is an ERP component elicited by rare “deviant” stimuli among repetitive “standard” stimuli over posterior electrode sites approximately at 100–200 ms post stimulus but also in a later latency range, up to 400 ms after the stimulus onset (e.g., Czigler et al., 2006; Astikainen et al., 2008; Stefanics et al., 2012, 2018; for a review, see Stefanics et al., 2014).

Related to depression, three studies have investigated the vMMN to changes in basic visual features (Chang et al., 2011; Qiu et al., 2011; Maekawa et al., 2013; for a review, see Kremláček et al., 2016), and one to changes in facial emotions (Chang et al., 2010). In study by Chang et al. (2010), centrally presented schematic faces were applied as stimuli (neutral faces as standard stimuli and happy and sad faces as deviant stimuli). The results showed that the early vMMN (reflecting mainly modulation of the N170 component) was reduced compared to the control group and the late vMMN (reflecting mainly modulation in P2 component) was absent in the depression group. This study thus demonstrated no negative bias, but a general deficit in the cortical change detection of facial expressions. Since in this study neutral faces were always applied as standard stimuli and emotional faces as deviant stimuli, it is unclear whether the modulations in ERPs were due to facial emotion processing as such or due to change detection in facial emotions. This applies nearly to all vMMN studies with facial expressions as the changing feature, as visual and face-sensitive components are known to be modulated by emotional expression (e.g., Batty and Taylor, 2003). This problem is particularly difficult when a neutral standard face and an emotional deviant face are used in the oddball condition, as the exogenous responses are the greatest to emotional faces (e.g., Batty and Taylor, 2003). This problem can be solved using only emotional faces in the oddball condition and analyzing the vMMN as a difference between the responses to the same facial emotion (e.g., a happy face) presented as deviant and standard stimuli (see Stefanics et al., 2012). This analysis method allows separating the vMMN, which reflects change detection, and the emotional modulation of visual and face-sensitive components.

In the present study, we investigated automatic face processing and change detection in emotional faces in two groups of participants: those with depressive symptoms (Beck’s Depression Inventory ≥ 13; here referred to as the dysphoric group) and in gender- and age- matched never-depressed control participants. The stimuli and procedure were similar to those reported previously by Stefanics et al. (2012), but instead of happy and fearful faces, we applied happy and sad faces. We chose happy and sad faces since impairments in the processing of both of these have been in previous studies associated to depression (happy faces: Fu et al., 2007; sad faces: e.g., Bradley et al., 1997; Gollan et al., 2008), and because these facial emotions make it possible to study mood-congruent negative bias in depression. Recordings of MEG were applied, which provide excellent temporal resolution and relatively good spatial resolution; in addition, its signal is less disturbed by the skull and scalp than the EEG signal (for a review, see Baillet, 2017).

Importantly, during the stimulus presentation the participants conducted a task related to stimuli presented in the center of the screen, while at the same time, emotional faces were presented in the periphery. In most of the previous studies of unattended face processing, face stimuli have been presented in the center of the visual field (e.g., Zhao and Li, 2006; Astikainen and Hietanen, 2009), as well as in the study where depression and control groups were compared (Chang et al., 2010). Centrally presented pictures might be difficult to ignore, and in real life, we also acquire information from our visual periphery. We hypothesize that rare changes in facial emotions presented in the peripheral vision in a condition in which participants ignore the stimuli will result in amplitude modulations in responses corresponding to the vMMN. We expect that the experimental manipulation of the stimulus probability will elicit the vMMN in three time windows reflecting the three stages of facial information processing. This hypothesis is based on previous ERP studies applying the oddball condition in which amplitude modulations in P100, N170, and P2 have been found (Zhao and Li, 2006; Astikainen and Hietanen, 2009; Chang et al., 2010; Susac et al., 2010; Stefanics et al., 2012). In addition to stimulus probability effects, modulations by facial emotions are expected in the MEG counterparts of N170 and P2 (Miyoshi et al., 2004; Zhao and Li, 2006; Japee et al., 2009; Chen et al., 2014; Hinojosa et al., 2015). However, it is not clear if the first processing stage, M100, can be expected to be different in amplitude for sad and happy faces. In ERP studies, the corresponding P1 component have been modulated in amplitude for happy and fearful faces (Luo et al., 2010; Stefanics et al., 2012), but there are no previous studies contrasting sad and happy face processing in a stimulus condition comparable to the present study. Importantly, based on prior studies we expect that a group difference can be found for vMMN at the time windows for P1, N170, and P2, but it might not be specific to sad or happy faces (Chang et al., 2010). A depression-related negative bias, larger responses to sad than happy faces specifically in the dysphoric group, is also expected. Studies that have used attended stimulus conditions suggest that the negative bias is present in the two later processing stages (i.e., N170 and P2, Dai and Feng, 2012; Chen et al., 2014; Zhao et al., 2015; Dai et al., 2016; also, for a negative bias in P1, see Dai and Feng, 2012).

## Materials and Methods

### Participants

Thirteen healthy participants (control group) and ten participants with self-reported depressive symptoms (dysphoric group) volunteered for the study. The participants were recruited via email lists and notice board announcements at the University of Jyväskylä and with an announcement in a local newspaper. Inclusion criteria for all participants were age between 18 and 45 years, right handedness, normal or corrected to normal vision, and no self-reported neurological disorders. Inclusion criteria for the participants in the dysphoric group were self-reported symptoms of depression (13 scores or more as measured with the BDI-II) or a recent depression diagnosis. The exclusion criteria for all participants were self-reported anamnesis of any psychiatric disorders other than depression or anxiety in the dysphoric group (such as bipolar disorder or schizophrenia) and current or previous abuse of alcohol or drugs.

All but one of the participants in the dysphoric group reported having a diagnosis of depression given by a medical doctor in Finland. According to their self-reported diagnoses, one participant had mild depression (F32.0), four had moderate depression (F32.1), one had severe depression (F32.2), two had recurrent depression with moderate episode (F33.1), and one did not remember which depression diagnosis was given. One participant reported to have a comorbid anxiety disorder, one reported a previous anxiety disorder diagnosis, and one reported a previous anxiety disorder combined with an eating disorder. They were included in the study because comorbidity with anxiety is high among depressed individuals. Because in some cases the diagnosis had been given more than 1 year ago, the current symptom level was assessed prior to the experiment with Beck’s Depression Inventory (BDI-II, Beck et al., 1996).

According to the BDI-II manual, the following normative cutoffs are recommended for the interpretation of BDI-II scores: 0–13 points = minimal depression, 14–19 points = mild depression, 20–28 points = moderate depression, and 29–63 points = severe depression (Beck et al., 1996). Based on these cut-off values, there were four participants with mild depression, four participants with moderate depression, and two participants with severe depression. The BDI-II scores and demographics are reported in **Table 1**. Written informed consent was obtained from the participants before their participation. The experiment was carried out in accordance with the Declaration of Helsinki. The ethical committee of the University of Jyväskylä approved the research protocol.

**Table 1:** Characteristics of the participants.

Variable		Group	
		Dysphoric	Control
*N*		10	13
Mean age (SD)		25.10 (4.51)	26.69 (7.65)
Level of education^a^	2	6	6
	3	4	7
Female/male		6/4	9/4
Time of diagnosis (within 6 months/within year/over a year ago		2/2/5	
Currently on psychotropic medication		6	0
Duration of antidepressant medication less than 1 year/1 year or more		2/4	
Antidepressant type		3 SSRIs, 3 SSRIs + bupropion^b^	
With a history of psychotropic medication		4	0
Currently in psychotherapy treatment for depression		2	0
With a history of psychotherapy		4	0
Currently have psychiatric diagnoses other than depression		1^c^	0
With a history of psychiatric diagnoses other than depression		2^d^	0
Mean BDI-II score (SD) [range]		22.40 (7.26) [13–36]	2.38 (2.40) [0–7]

*^a^Levels of education were coded as follows: 2 = middle level (high school or equivalent); 3 = high level (bachelor or higher degree). ^b^SSRIs, selective serotonin reuptake inhibitors. ^c^One of the participants also suffered from anxiety at the time of the study. ^d^Two of the participants had a self-reported previous anxiety disorder, and one of them was diagnosed with anorexia in youth.*

### Stimuli and Procedure

The visual stimuli were black and white photographs (3.7° wide × 4.9° tall) of 10 different models (five males and five females) from Pictures of Facial Affect (Ekman and Friesen, 1976). Stimuli were presented on a dark-gray background screen at a viewing distance of 100 cm. Each trial consisted of four face stimuli randomly presented at four fixed locations at the corners of an imaginary square (eccentricity, 5.37°) and a fixation cross in the center of the screen. The four faces were presented at the same time, each face showing the same emotion (either happy or sad). On each panel, two male and two female faces were presented. The duration of each stimulus was 200 ms.

An oddball condition was applied in which an inter-stimulus interval (ISI) randomly varied from 450 to 650 ms (offset to onset). The experiment consisted of four stimulus blocks in which frequent (90%; standard) stimuli were randomly interspersed with rare (10%; deviant) stimuli. In two experimental blocks, sad faces were presented frequently as standard stimuli, while happy faces were presented rarely as deviant stimuli. In the other two blocks, happy faces were presented as standard stimuli and sad faces were presented as deviant stimuli. Each block contained 450 standard stimuli and 50 deviant stimuli, and the order of the four blocks were randomized across participants.

The participants’ task was to fixate to the cross in the center of the screen, ignore the emotional faces, and respond by pressing a button as soon as possible when they detected a change of the cross in the screen center. The change in cross was a lengthening of its horizontal line or vertical line with a frequency of 11 changes per minute. Face and cross changes never co-occurred.

### Data Acquisition

The visually evoked magnetic fields were recorded with a 306-channel whole-head system (Elekta Neuromag Oy, Helsinki, Finland) consisting of 204 planar gradiometers and 102 magnetometers in a magnetically shielded room at the MEG Laboratory, University of Jyväskylä. The empty room activity was recorded for 2 min before and after the experiment to estimate intrinsic noise levels. It was confirmed that all the magnetic materials that may distort the measurement had been removed from participants before the experiment. The locations of three anatomical landmarks (the nasion and left and right preauricular points) and five Head Position Indicator coils (HPI-coils, two on the forehead, two behind the ears, and one on the crown), as well as a number of additional points on the head were determined with an Isotrak 3D digitizer (Polhemus^TM^, United States) before the experiment started. During the recording, participants were instructed to sit in a chair with their head inside the helmet-shaped magnetometer and their hands on a table. The vertical electro-oculogram (EOG) was recorded with bipolar electrodes, one above and one below the right eye. The horizontal EOG was recorded with bipolar electrodes placed lateral to the outer canthi of the eyes.

### Data Analysis

#### MEG Data

First, the spatiotemporal signal space separation (tSSS) method (Taulu et al., 2005; Supek and Aine, 2014) in the MaxFilter software (Elekta-Neuromag) was used to remove the external interference from the MEG data. The MaxFilter software was also applied for head movement correction and transforming the head origin to the same position for each participant. Then, the MEG data were analyzed using the Brainstorm software (Tadel et al., 2011). Recordings were filtered offline by a band-pass filter between 0.1 and 40 Hz. To avoid potential artifacts, epochs with values exceeding ±200 μV in EOG channels were rejected from the analysis. Next, eye blink and heartbeat artifacts were identified based on EOG and electrocardiographic (ECG) channels using a signal-space projection (SSP) method (Uusitalo and Ilmoniemi, 1997) and removed from the data. To compare the results more directly with the previous ERP studies and to the results of Stefanics et al. (2012) in particular, data from magnetometers were analyzed. The data were segmented into epochs from -200 ms before to 600 ms after the stimulus onset and baseline corrected to the 200 ms pre-stimulus period. Trials were averaged separately for happy standard, sad standard, happy deviant, and sad deviant stimuli for each participant, the number of accepted trials being 651 (*SD* = 13.57), 639 (*SD* = 23.45), 83 (*SD* = 3.18), and 81 (*SD* = 4.69), respectively. The percentage of accepted trials for happy and sad deviants, and happy and sad standards were 83% (*SD* = 3.18%), 81% (*SD* = 4.69%), 72% (*SD* = 1.51%), and 71% (*SD* = 2.61%), respectively. There were no group differences in the number of accepted trials (all *p*-values > 0.34).

**FIGURE 1:**
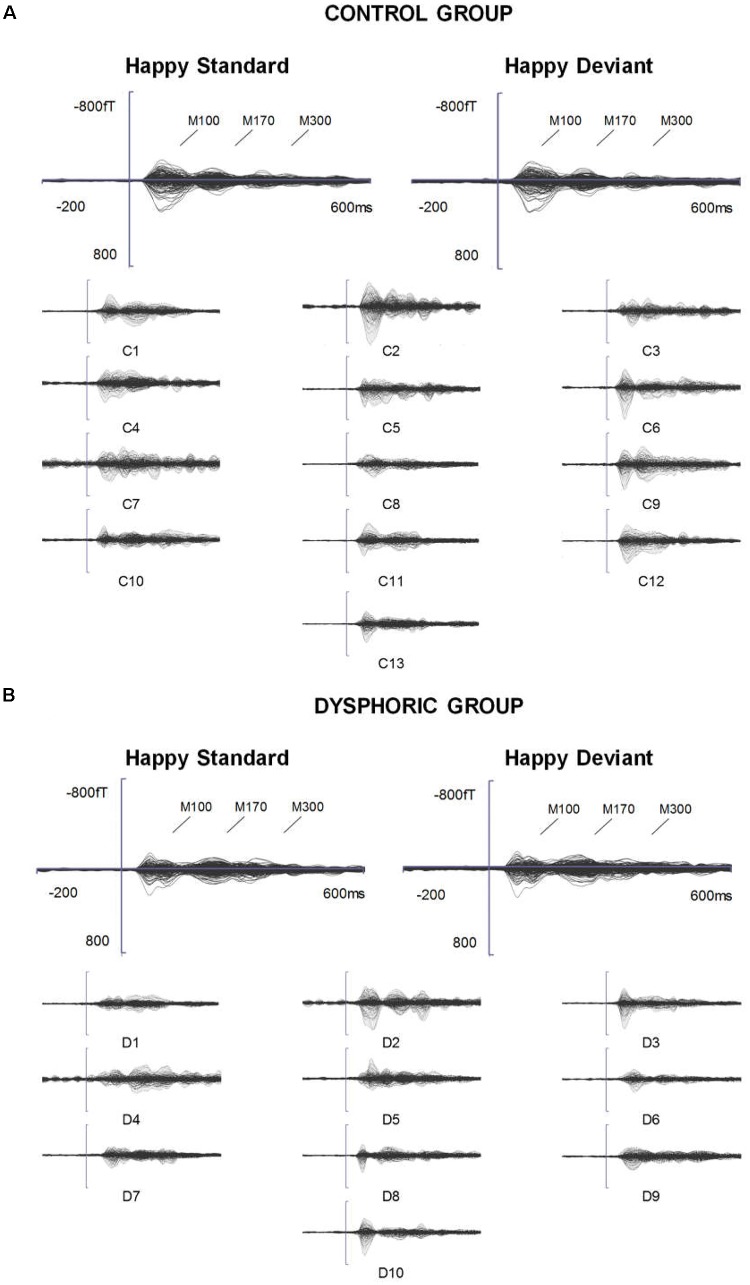
ERFs reflecting three stages of face processing (M100, M170, and M300). **(A)** Butterfly view of the grand-averaged responses to happy standard and happy deviant faces in the control group (top) and individual participants’ responses to happy standard faces (C1–C13). **(B)** Butterfly view of the grand-averaged responses to happy standard and happy deviant faces in the dysphoric group (top) and individual participants’ responses to happy standard faces (D1–D10). It should be noted that signals from both magnetometers and gradiometers are included here. For visualization purposes, the gradiometer values are 0.04 times multiplied due to the different units for magnetometers (T) and gradiometers (T/m).

**FIGURE 2:**
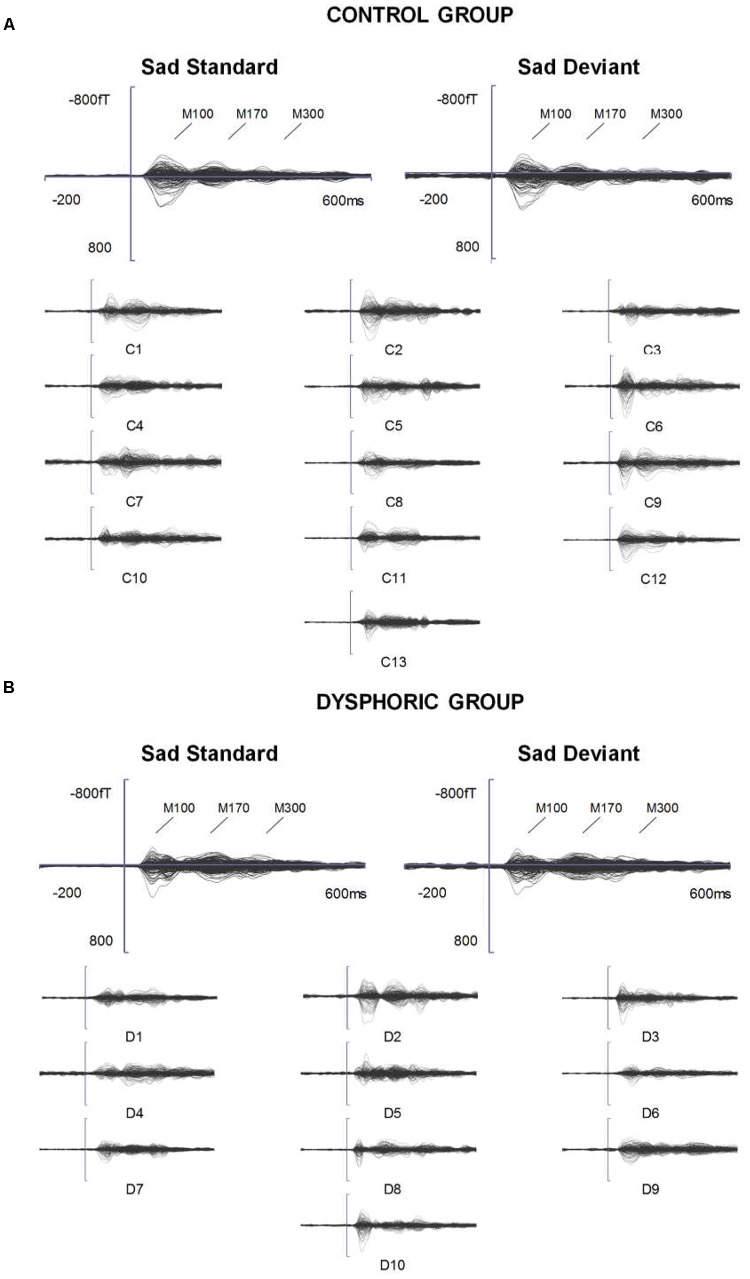
ERFs reflecting three stages of face processing (M100, M170, and M300). **(A)** Butterfly view of the grand-averaged responses to sad standard and sad deviant faces in the control group (top) and individual participants’ responses to sad standard faces (C1–C13). **(B)** Butterfly view of the grand-averaged responses to sad standard and sad deviant faces in the dysphoric group (top) and individual participants’ responses to sad standard faces (D1–D10). It should be noted that signals from both magnetometers and gradiometers are included here. For visualization purposes, the gradiometer values are 0.04 times multiplied due to the different units for magnetometers (T) and gradiometers (T/m).

**FIGURE 3:**
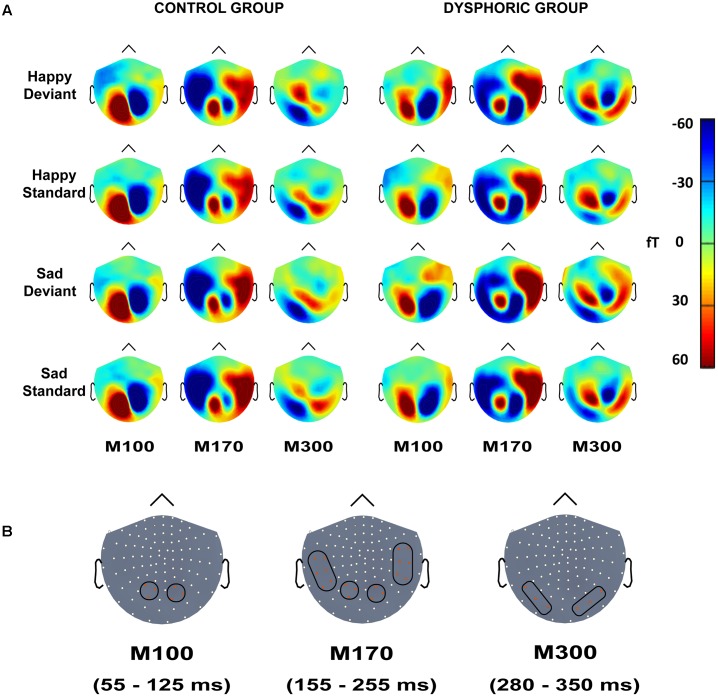
**(A)** Topographic maps of grand-averaged magnetic fields for happy deviant, happy standard, sad deviant, and sad standard faces in the control (left) and dysphoric groups (right) for the M100, M170, and M300 shown here at 85, 205, and 315 ms after stimulus onset, respectively. **(B)** Magnetometer sensors and the regions of interests used for analyses are marked with black frames for M100, M170, and M300. Each participant’s peak values were extracted from the time windows of 55–125 ms, 155–255 ms, and 280–350 ms after stimulus onset for M100, M170, and M300, respectively.

The peak amplitude values for each participant, separately for each stimulus type and emotion, were measured in three time windows: 55–125 ms, 155–255 ms, and 280–350 ms post-stimulus, corresponding to the three major responses, M100, M170, and M300, found from the grand-averaged data (**Figures 1**–**3**). Based on prior findings (Peyk et al., 2008; Taylor et al., 2011; Stefanics et al., 2012), we defined two (M100, M300) or four (M170) regions of interest (ROIs) for the peak amplitude analysis for each response (**Figure 3**). For M100, the peak amplitudes were averaged across sensors at bilateral occipital regions (Left ROI: MEG1911, MEG1921, MEG2041; Right ROI: MEG2311, MEG2321, MEG2341). For M170, the peak amplitudes were averaged across sensors at bilateral temporal and occipital sites (Left temporal ROI: MEG1511, MEG1521, MEG1611, MEG1641, MEG1721, MEG0241; Right temporal ROI: MEG1321, MEG1331, MEG1441, MEG2421, MEG2611, MEG2641; Left occipital ROI: MEG1911, MEG1921, MEG2041; Right occipital ROI: MEG2311, MEG2321, MEG2341). For M300, the peak amplitude values were averaged across sensors at occipital sites (Right ROI: MEG1721, MEG1731, MEG1931; Left ROI: MEG2331, MEG2511, MEG2521). In addition to peak amplitudes, the peak latencies were also measured for each component from the same sensors as used in the amplitude analysis. Since two participants’ data did not show M300 responses (one in control group and another in dysphoric group), they were excluded from the analysis for this response.

#### Behavioral Data

For the behavioral task, the analysis included hit rate and false alarm calculations. The hit rate was calculated as the ratio between button presses in a 100–2,000 ms interval after the event and the actual number of cross-changes. The false alarm rate was calculated as the ratio between button presses that were not preceded by a cross-change in a 100–2,000 ms interval before the event and the actual number of cross-changes.

### Statistical Analyses

Repeated-measures analysis of variance (ANOVA) was used to analyze the reaction time and accuracy (hit rate and false alarm) for the cross-change task. A within-subjects factor Stimulus Block (Sad vs. Happy standard) and a between-subjects factor Group (Control vs. Dysphoric) were applied.

Peak amplitudes and peak latencies separately at different ROIs in three time windows were analyzed with a three-way repeated-measures ANOVA with within-subjects factors Stimulus Type (Standard vs. Deviant) and Emotion (Sad vs. Happy), and a between-subjects factor Group (Control vs. Dysphoric).

In addition, because the visual inspection of the topographic maps of the M300 showed that there might be differences in the lateralization of the responses in dysphoric and control groups, we further studied this possibility using the lateralization index. First, all peak values from the right hemisphere ROI were multiplied by -1 to correct for the polarity difference (also see Morel et al., 2009; Ulloa et al., 2014). Then, using these rectified response values, the lateralization index was calculated for responses to each stimulus type (Happy Deviant, Happy Standard, Sad Deviant, Sad Standard) as follows: Lateralization index = (Left – Right)/(Left + Right). A three-way repeated-measures ANOVA with the within-subjects factors Stimulus Type (Standard vs. Deviant) and Emotion (Sad vs. Happy), and the between-subjects factor Group (Control vs. Dysphoric) was applied.

Besides, possible differences in the lateralization of the M300 response were investigated separately for happy and sad, as well as for deviant, and standard stimulus responses with repeated-measures ANOVAs. Furthermore, because small sample size can limit the possibility to observe existing significant differences in multi-way ANOVAs, we also compared lateralization indexes separately for the happy (Deviant happy – Standard happy) and sad (Deviant sad – Standard sad) vMMN between the groups with independent samples *t*-tests (bootstrapping method with 1,000 permutations; Supplementary Materials).

For all significant ANOVA results, *post hoc* analyses were conducted using two-tailed paired *t*-tests to compare the differences involving within-subjects factors and using independent-samples *t*-tests for between-subjects comparisons, both with a bootstrapping method using 1,000 permutations (Good, 2005).

For all analyses, $\eta_{p}^{2}$ presents effect size estimates for ANOVAs and Cohen’s d for *t*-tests. Cohen’s d was computed using pooled standard deviations (Cohen, 1988). In addition, we conducted the Bayes factor analysis to estimate whether the null results in *post hoc* analyses were observed by chance (Rouder et al., 2009). The Bayes Factor (*BF_10_*) provides an odds ratio for the alternative/null hypotheses (values < 1 favor the null hypothesis and values > 1 favor the alternative hypothesis). For example, a *BF_10_* of 0.5 would indicate that the null hypothesis is two times more likely than the alternative hypothesis.

Whenever a significant interaction effect with the factor Group was found, two-tailed Pearson correlation coefficients were used to evaluate the correlation between the BDI-II score and the brain response. Bootstrap estimates of correlation were performed with 1,000 permutations.

The significance level was set to *p* < 0.05 for all tests.

## Results

### Reaction Time and Hit Rate

For the reaction time and accuracy, neither significant main effects nor interaction effects were found (all *p*-values > 0.17). The mean reaction times were 384 ms (*SD* = 53) and 394 ms (*SD* = 69) for happy and sad standard stimulus blocks, respectively, and the mean reaction time for the whole experiment was 386 ms (*SD* = 61). The hit rate for blocks with happy faces as the standard stimuli was 98.86% (*SD* = 0.02), and 98.74% (*SD* = 0.02) for blocks with sad standard faces. The mean hit rate was 98.79% (*SD* = 0.02) for the whole experiment. The mean false alarm was below 1% both for happy and sad standard stimulus blocks, and the mean of the experiment was 0.96% (*SD* = 0.01). The mean reaction times were 380 ms (*SD* = 16) and 393 ms (*SD* = 27) for the control and dysphoric groups, respectively. The hit rate was above 98% for both groups (*M* = 99.1%, *SD* = 0.02 for the control group; *M* = 98.4%, *SD* = 0.02 for the dysphoric group). The mean false alarm rates were 0.978% (*SD* = 0.01) for the control group and 0.931% (*SD* = 0.004) for the dysphoric group, respectively.

### Evoked Magnetic Fields

The grand-averaged evoked fields showed characteristic M100, M170, and M300 responses for both happy and sad faces presented as standard and deviant stimuli (**Figures 1**, **2**). Butterfly views of the standard and deviant responses and each individual participant’s responses (for the standards only) are shown separately for happy (**Figure 1**) and sad stimuli (**Figure 2**) for the control and dysphoric groups. The topographic maps for each response type are shown in **Figure 3A**, and the ROIs for each response are shown in **Figure 3B**.

The response latencies are reported in **Table 2**. There were no significant main effects or interaction effects in response latencies.

**Table 2:** Mean peak latency (ms) and standard deviation (in parentheses) for each response and ROI in the control and dysphoric groups.

Response	ROI	Group	Happy Deviant	Happy Standard	Sad Deviant	Sad Standard
M100	Left occipital	Con	81.15	82.69	81.15	78.85
			(15.02)	(20.88)	(19.38)	(14.46)
		Dys	82.00	86.00	86.00	80.00
			(21.63)	(24.70)	(23.31)	(20.68)
	Right occipital	Con	82.69	83.46	85.00	84.23
			(22.04)	(21.54)	(20.82)	(22.16)
		Dys	85.00	79.00	78.00	87.00
			(22.61)	(22.71)	(23.12)	(23.94)
M170	Left temporal	Con	197.31	205.77	205.00	197.31
			(29.20)	(27.53)	(28.58)	(19.64)
		Dys	204.00	208.00	202.00	195.00
			(34.14)	(33.68)	(37.43)	(22.11)
	Right temporal	Con	198.08	205.77	199.62	188.85
			(23.59)	(28.13)	(26.96)	(22.93)
		Dys	207.00	212.00	210.00	208.00
			(21.50)	(29.08)	(32.06)	(30.57)
	Left occipital	Con	205.00	198.85	209.62	202.69
			(35.36)	(36.18)	(39.08)	(38.11)
		Dys	213.00	210.00	216.00	209.00
			(29.74)	(33.75)	(34.79)	(35.96)
	Right occipital	Con	220.38	222.69	221.15	228.85
			(26.34)	(22.04)	(22.93)	(26.94)
		Dys	220.00	201.00	218.00	209.00
			(25.90)	(35.34)	(28.30)	(32.04)
M300	Left occipital	Con	312.50	319.17	325.00	319.17
			(22.56)	(22.79)	(25.12)	(27.74)
		Dys	320.56	312.78	317.22	315.00
			(21.67)	(14.14)	(18.47)	(17.53)
	Right occipital	Con	322.50	315.00	317.50	315.83
			(26.65)	(26.19)	(27.69)	(25.12)
		Dys	316.11	302.78	307.22	309.44
			(22.68)	(17.73)	(16.04)	(13.89)

*Please not that there were no significant main or interaction effects. ROI, region of interest; Con, control group; Dys, dysphoric group.*

The peak amplitude values are reported in **Table 3**. Next, the results of the amplitude analyses are reported separately for each component.

**Table 3:** Peak amplitude values (fT) and standard deviation (in parentheses) for each response and ROI in the control and dysphoric groups.

Response	ROI	Group	Happy Deviant	Happy Standard	Sad Deviant	Sad Standard
M100	Left occipital	Con	205.01	197.65	204.25	208.82
			(106.34)	(106.50)	(101.10)	(105.65)
		Dys	177.40	176.86	170.18	174.31
			(88.01)	(96.45)	(89.65)	(95.29)
	Right occipital	Con	-215.75	-195.07	-213.29	-195.99
			(104.50)	(101.15)	(96.33)	(95.12)
		Dys	-196.74	-163.74	-187.07	-175.14
			(102.74)	(129.88)	(108.73)	(117.40)
M170	Left temporal	Con	-98.35	-92.92	-104.55	-99.67
			(69.37)	(68.64)	(72.84)	(71.37)
		Dys	-77.75	-80.88	-85.04	-89.26
			(59.75)	(42.71)	(49.25)	(50.22)
	Right temporal	Con	70.11	62.36	81.88	77.53
			(44.29)	(42.32)	(51.03)	(50.58)
		Dys	86.32	85.65	98.39	96.24
			(52.82)	(54.90)	(64.58)	(57.11)
	Left occipital	Con	112.17	97.80	107.91	86.69
			(73.62)	(70.38)	(66.50)	(88.80)
		Dys	95.66	94.75	99.89	85.99
			(43.34)	(49.98)	(47.07)	(44.23)
	Right occipital	Con	-94.81	-76.31	-90.09	-67.80
			(103.98)	(89.04)	(83.11)	(99.68)
		Dys	-130.14	-119.41	-125.80	-114.16
			(57.09)	(51.14)	(53.25)	(60.40)
M300	Left occipital	Con	-80.66	-76.89	-77.79	-74.09
			(37.14)	(53.74)	(49.35)	(57.56)
		Dys	-54.96	-64.29	-78.27	-57.78
			(55.79)	(53.48)	(55.06	(54.06)
	Right occipital	Con	51.04	65.13	48.29	67.49
			(31.33)	(38.01)	(39.16)	(37.67)
		Dys	50.18	58.38	57.58	49.77
			(38.65)	(49.73)	(59.23)	(35.95)

*ROI, region of interest; Con, control group; Dys, dysphoric group.*

#### M100

**FIGURE 4:**
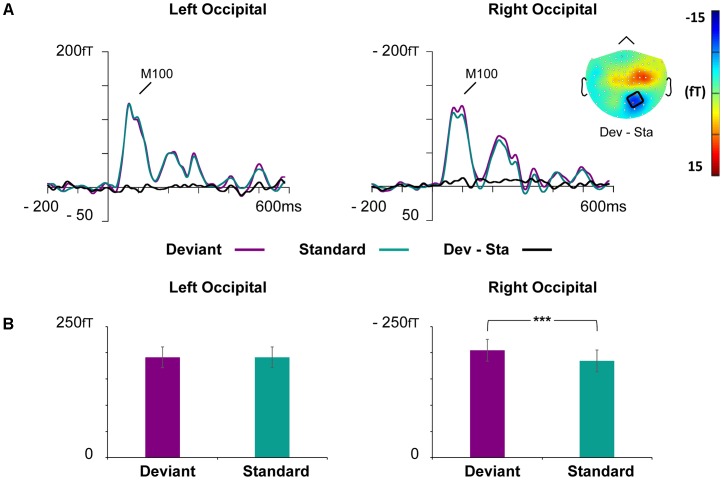
Grand-averaged ERFs demonstrating the M100. **(A)** Waveforms of evoked magnetic fields to the standard and deviant stimuli and deviant minus standard differential response at the left and right occipital ROIs. The topography of the vMMN (deviant – standard) depicted as the mean value of activity 55–125 ms after stimulus onset. **(B)** Bar graph for the M100 peak value with standard errors to deviant and standard faces at the left and right occipital ROIs. Asterisks mark a significant difference at ^∗∗∗^*p* < 0.001.

Waveforms of the event-related magnetic fields (ERFs) showed a strong M100 response peaking approximately at 85 ms after the stimulus onset on bilateral occipital regions (**Figure 4**).

At the left occipital ROI, neither main effects nor interaction effects were found (all *p*-values > 0.226).

At the right occipital ROI, there was a significant main effect of Stimulus Type, *F*(1,21) = 30.22, *p* < 0.001, $\eta_{p}^{2}$ = 0.59, indicating larger ERF amplitudes for the deviant faces than standard faces. The other main effects and all interaction effects were non-significant (all *p*-values > 0.342).

#### M170

**FIGURE 5:**
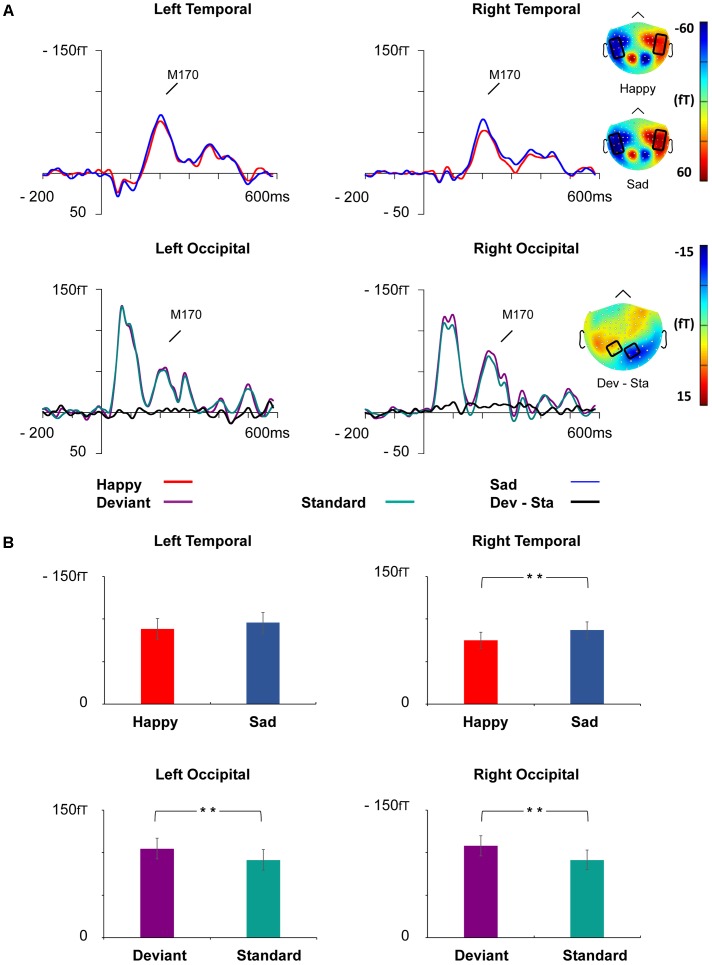
Grand-averaged ERFs demonstrating the M170 response. **(A)** Upper: Waveforms of ERFs to happy and sad faces at right and left temporal ROIs and corresponding topographic maps. Lower: Waveforms of ERFs to standard and deviant faces at the left and right occipital ROIs and the topography of the vMMN (deviant – standard). Topographic maps are depicted as the mean value of activity 155–255 ms after stimulus onset. **(B)** Bar graph for the M170 peak amplitude values with standard errors to happy and sad faces (upper) and deviant and standard stimuli (lower) at the left and right temporal and occipital ROIs, respectively. Asterisks mark significant differences at ^∗∗^*p* < 0.01.

Event-related magnetic field waveforms showed a strong M170 response peaking approximately 205 ms after the stimulus onset in the bilateral temporo-occipital regions (**Figure 5**).

At the left temporal ROI, there was a marginally significant main effect for Emotion, *F*(1,21) = 3.46, *p* = 0.077, $\eta_{p}^{2}$ = 0.14, reflecting more activity for sad than happy faces. Other main effects and interaction effects were non-significant (all *p*-values > 0.261).

At the right temporal ROI, a main effect of Emotion was observed, *F*(1,21) = 8.52, *p* = 0.008, $\eta_{p}^{2}$ = 0.29, wherein sad faces induced larger amplitudes than happy faces. Neither other main effects nor any of the interaction effects were significant (all *p*-values > 0.353).

At the left occipital ROI, a main effect of Stimulus Type was found, *F*(1,21) = 9.29, *p* = 0.006, $\eta_{p}^{2}$ = 0.31, reflecting larger activity for deviant faces than standard faces. Other main effects and interaction effects were non-significant (all *p*-values > 0.223).

At the right occipital ROI, a main effect of Stimulus Type was found, *F*(1,21) = 12.81, *p* = 0.002, $\eta_{p}^{2}$ = 0.38, reflecting larger activity for deviant faces than standard faces. Other main effects and interaction effects were non-significant (all *p*-values > 0.288).

#### M300

##### Amplitude results

**FIGURE 6:**
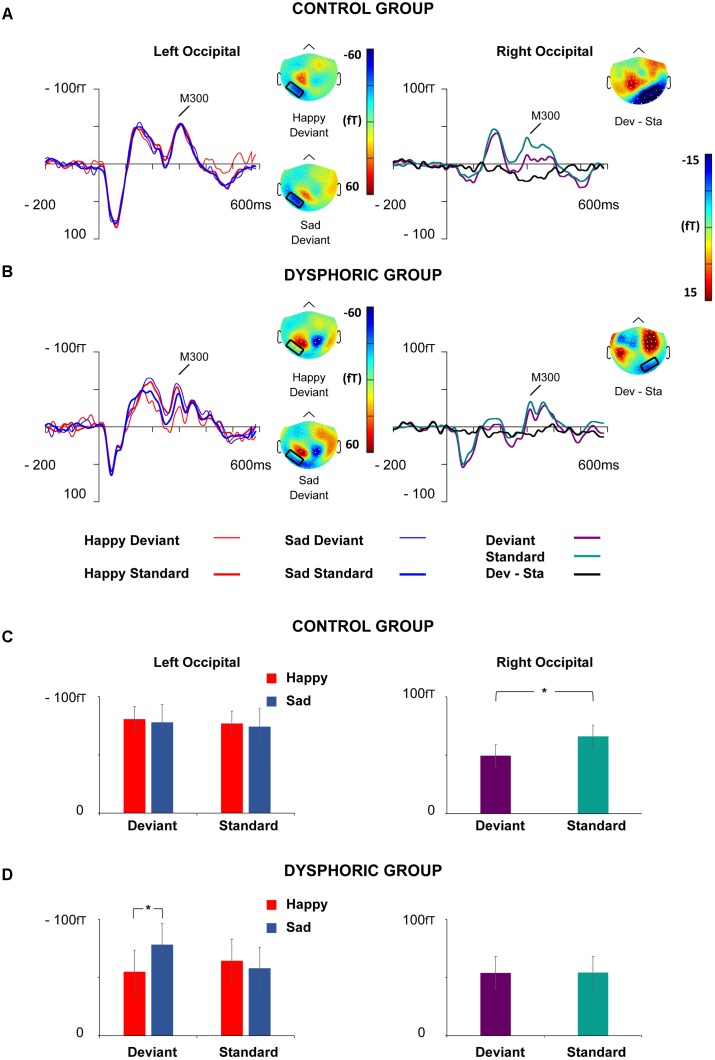
Grand-averaged ERFs for the M300 response. Waveforms of ERFs at the left and right occipital ROIs in the control **(A)** and dysphoric group **(B)**. Topographic maps of Happy and Sad Deviant (Left), and the vMMN (Deviant – Standard) responses (Right) are depicted as the mean value of activity 280–350 ms after stimulus onset. Bar graphs for the M300 peak value with standard errors at left and right occipital ROIs in the control **(C)** and dysphoric group **(D)**. Note that in the left hemisphere there was an interaction effect of Emotion × Stimulus Type × Group, which showed that sad deviant faces induced more activity than happy deviant faces in the dysphoric group but not in the control group. In the right hemisphere, an interaction effect of Stimulus Type × Group was found, indicating that the vMMN was elicited in the control but not in the dysphoric group. Asterisk marks significant differences at ^∗^*p* < 0.05.

The waveforms of the ERF demonstrated a bipolar M300 activity over the bilateral occipital ROI peaking approximately 315 ms after the stimulus onset (**Figure 6**).

At the left occipital ROI, a significant interaction effect of Emotion × Stimulus Type, *F*(1,19) = 4.48, *p* = 0.048, $\eta_{p}^{2}$ = 0.19, a marginally significant interaction effect of Emotion × Group, *F*(1,19) = 4.34, *p* = 0.051, $\eta_{p}^{2}$ = 0.186, and a significant interaction effect of Emotion × Stimulus Type × Group, *F*(1,19) = 4.52, *p* = 0.047, $\eta_{p}^{2}$ = 0.19, was found. The other main effects and interaction effects were non-significant (all *p*-values > 0.315).

*Post hoc* tests for the Emotion × Stimulus Type interaction did not show any significant differences for any of the comparisons (all *p*-values > 0.128, all *BF_10_*s < 0.67).

*Post hoc* tests for Emotion × Group interaction showed that sad faces induced larger activity than happy faces in the dysphoric group, *t*(8) = 3.27, *p* = 0.030, CI 95% [3.72, 13.34], *d* = 0.16, *BF_10_* = 5.72, but there were no differences between the responses to sad and happy faces in the control group, *t*(12) = 0.67, *p* = 0.513, CI 95% [-11.14, 4.85], *d* = 0.06, *BF_10_* = 0.35. No differences between the groups were found in happy or sad face responses (all *p*-values > 0.395, all *BF_10_*s < 0.53).

*Post hoc* tests for the three-way interaction showed that no differences were found between the groups in amplitudes to any of the stimulus types *per se* (Happy Deviant, Happy Standard, Sad Deviant, Sad Standard, all *p*-values > 0.244, all *BF_10_*s < 0.68), or in the vMMN responses (Happy Deviant – Happy Standard, Sad Deviant – Sad Standard), all *p*-values > 0.225, all *BF_10_*s < 0.67). Thus, we split the data by group and run a two-way repeated-measures of ANOVA with Stimulus Type (Standard vs. Deviant) × Emotion (Sad vs. Happy) in each group separately. There was neither a significant main effect nor interaction effects in the control group (all *p*-values > 0.517). In the dysphoric group, an interaction effect of Emotion × Stimulus type was found, *F*(1,8) = 6.87, *p* = 0.031, $\eta_{p}^{2}$ = 0.46. Amplitude values for sad deviant faces were larger than for happy deviant faces, *t*(8) = 4.91, *p* = 0.011, CI 95% [15.18, 32.98], *d* = 0.38, *BF_10_* = 35.9. Further, a marginally significant difference was found reflecting larger amplitude values for sad deviant faces than sad standard faces in the dysphoric group, *t*(8) = 2.09, *p* = 0.085, CI 95% [-38.45, -1.19], *d* = 0.38, *BF_10_* = 1.42. No other significant results between responses to different stimulus type pairs were found in the dysphoric group (all *p*-values > 0.168, all *BF_10_*s < 0.76). There was also a main effect of Emotion in the dysphoric group, *F*(1,8) = 10.67, *p* = 0.011, $\eta_{p}^{2}$ = 0.57, reflecting more activity for sad faces than happy faces.

At the right occipital ROI, the main effect of the Stimulus Type, *F*(1,19) = 5.40, *p* = 0.031, $\eta_{p}^{2}$ = 0.22, and an interaction effect of Stimulus Type × Group was found, *F*(1,19) = 5.15, *p* = 0.035, $\eta_{p}^{2}$ = 0.21. The other main effects and interaction effects were non-significant (all *p*-values > 0.328).

The responses were smaller in amplitude for deviant faces than for standard faces in the whole group level. *Post hoc* analysis for the Stimulus Type × Group interaction revealed that the groups did not differ in any of the stimulus responses as such (all *p*-values > 0.476, all *BF_10_*s < 0.48). However, in the control group responses to deviant faces were smaller in amplitude than those to standard faces, *t*(11) = 2.87, *p* = 0.020, CI 95% [-28.14, -5.97], *d* = 0.49, *BF_10_* = 4.23. There was no such difference in the dysphoric group, *t*(8) = 0.06, *p* = 0.949, CI 95% [-6.29, 6.02], *d* = 0.005, *BF_10_* = 0.32. In addition, a group difference was found in the vMMN amplitude (deviant – standard differential response), *t*(19) = 2.27, *p* = 0.031, CI 95% [-28.86, -4.01], *d* = 1.05, *BF_10_* = 2.14, reflecting a larger vMMN amplitude in the control than in the dysphoric group.

##### Correlation analysis

In the whole group level the correlations between BDI-II scores and M300 response amplitudes were non-significant for all stimulus types at the left (all *p*-values > 0.062) and right occipital ROIs (all *p*-values > 0.438). The same applied for the correlations calculated separately for the dysphoric group (at the left ROI, all *p*-values > 0.107 and at the right ROI, all *p*-values > 0.299).

##### Lateralization index

The analysis of the lateralization index revealed neither main effects nor interaction effects (all *p*-values > 0.107).

## Discussion

The main goal of the present study was to examine the emotional encoding and automatic change detection of peripherally presented facial emotions in dysphoria. MEG recordings showed prominent M100, M170, and M300 components to emotional faces. All of the components were modulated by the presentation rate of the stimulus (deviant vs. standard), corresponding to the results of the previous studies conducted on healthy participants (Zhao and Li, 2006; Astikainen and Hietanen, 2009; Susac et al., 2010; Stefanics et al., 2012). M170 was also modulated by emotion, responses being larger for sad than happy faces. M300 showed both a negative bias and impaired change detection in dysphoria.

The negative bias in the dysphoric group, which was demonstrated as a relative difference in M300 amplitude for sad and happy faces in comparison to the control group, seems to be associated with change detection, as the deviant stimulus responses, but not the standard stimulus responses, were larger for sad than happy faces in the dysphoric group. This is a novel finding, and previous studies using the oddball condition in depressed participants have not separately investigated responsiveness for standard and deviant stimuli but used the differential response (deviant – standard) in their analysis (Chang et al., 2010, 2011; Qiu et al., 2011). In general, our finding of the negative bias in emotional face processing extends from the previous findings involving attended stimulus conditions (Bistricky et al., 2014; Chen et al., 2014; Zhao et al., 2015; Dai et al., 2016) to ignore condition. However, the negative bias in the present study was not found in the first processing stages (M100 and M170) as in the studies applying attentive condition in depressed participants (Dai and Feng, 2012; Chen et al., 2014; Zhao et al., 2015). In a prior study with dysphoric participants, elevated P3 ERP component to sad target faces reflected negative bias in attentive face processing in previously depressed participants, but no differences were found in the earlier N2 component in comparison to never depressed participants (Bistricky et al., 2014). Future studies are needed to investigate whether the discrepancy between our results and previous studies with depressed participants (Dai and Feng, 2012; Chen et al., 2014; Zhao et al., 2015) is related to different participant groups (depressed vs. dysphoric) or amount of attention directed toward stimuli (ignore vs. attend condition).

The finding that control participants, but not dysphoric participants, showed the vMMN in the right occipital region indicates that, in addition to the negative bias, there is a deficit in change detection in general in dysphoria. Our results thus reveal that a dysphoric state affects not only attended change detection in facial emotions (e.g., Bistricky et al., 2014; Chen et al., 2014), but also the automatic change detection of emotional faces in one’s visual periphery. This finding of a vMMN deficit in dysphoria at the latency window of the M300 is also in line with the previous ERP study applying an ignore condition and reporting that the late vMMN (at 220–320 ms, reflecting the modulation of P2) was observed in the control group but was absent in the depression group (Chang et al., 2010). However, since in this study standard faces were neutral and deviant faces emotional, the effects of emotional processing and deviance detection cannot be distinguished. Here, we calculated the vMMN as a differential response between the responses to the same stimulus presented as deviant and standard. Our results showing the decreased vMMN amplitude at the latency of M300 in the dysphoric group, relative to the control group, indicate that it is specifically the change detection that is impaired in participants with depression symptoms.

Here, we did not find group differences in the vMMN related to earlier processing stages. The previous vMMN study conducted on depressed participants has reported a larger vMMN at the latency of N170 in control than depression group, and also larger vMMN amplitude to sad than happy deviant faces (Chang et al., 2010). However, again, it is unclear whether these findings reflect emotional encoding or deviance detection, as in this study standard faces were neutral and deviant faces emotional and the vMMN was calculated as a difference between responses to these.

Our finding of the altered emotional vMMN in dysphoria is in line with prior results in schizophrenia, a psychiatric disorder with known deficits in emotion processing, in which diminished automatic brain responses to emotional faces in patients were reported (Csukly et al., 2013). The vMMN was also suggested in autism spectrum disorder as an indicator of affective reactivity; given that vMMN responses to emotional faces showed a correlation with Autism Spectrum Quotient (AQ) scores (Gayle et al., 2012). Here, we did not find correlations between the M300 amplitude and BDI-II scores within the dysphoric group. The lack of correlations can be interpreted as indicating that the alterations observed in M300 reflect more trait- than state-dependent factors of depression. However, the lack of correlation can also be explained by the small sample size.

We also investigated the possible lateralization effect for the occipital M300 because the visual observation of the topographical maps showed some differences between the groups in lateralization for this component. However, none of the various investigations revealed differences in the lateralization between the groups. There were no clear lateralization differences in M300 in sad and happy face processing in the whole group level either. Some previous studies have reported that the vMMN to emotional faces has a right hemisphere dominance (Gayle et al., 2012; Li et al., 2012; Stefanics et al., 2012), while others have not found it (Kovarski et al., 2017), but these findings have been related to earlier face processing stages.

Besides the findings related to dysphoria, there were findings related to automatic change detection and emotion processing that apply to the whole participant group. All investigated components (M100, M170, and M300) were modulated by stimulus rarity, likely reflecting the vMMN response. In the previous EEG and MEG studies, the vMMN has been elicited at the earliest processing stage, i.e., in the P1 time window (Susac et al., 2010; Stefanics et al., 2012) but also at the latency of N170 and later P2 component (Zhao and Li, 2006; Astikainen and Hietanen, 2009; Chang et al., 2010). It should be noted that it is unclear whether the vMMN to emotional faces is a separate component from the visual and face-related components (i.e., P1, N170, and P2) or whether the vMMN is the amplitude modulation of these canonical components. To our knowledge, only one previous study has directly addressed this question. In this study, independent component analysis (ICA) and two stimulus conditions varying the probability of the emotional faces were used to separate vMMN and N170 components (Astikainen et al., 2013). The ICA revealed two components within the relevant 100–200 ms latency range. One component, conforming to N170, differed between the emotional and neutral faces, but not as a function of the stimulus probability, and the other, confirming to vMMN, was also modulated by the stimulus probability. However, neither in this study (Astikainen et al., 2013) nor in other previous studies the functional independence of the vMMN from P1/M100 or P2/M300 responses have been investigated.

Here emotional modulation was found at the second stage (M170) of face processing, as in several previous studies (Batty and Taylor, 2003; Eger et al., 2003; Williams et al., 2006; Zhao and Li, 2006; Blau et al., 2007; Leppänen et al., 2007; Schyns et al., 2007; Japee et al., 2009; Wronka and Walentowska, 2011; Astikainen et al., 2013; Chen et al., 2014). The emotional modulation of M170 was observed at the right temporal ROI, which corresponds to previous findings (Williams et al., 2006; Japee et al., 2009; Wronka and Walentowska, 2011). In our study, sad faces induced a greater N170 response than happy faces, while previous ERP studies have not found a difference between the N170 amplitude for happy and sad faces (Batty and Taylor, 2003; Hendriks et al., 2007; Chai et al., 2012). It is notable, however, that in the present study the involvement of dysphoric participants might explain the difference in the results compared to previous studies conducted only on healthy participants (Batty and Taylor, 2003; Hendriks et al., 2007; Chai et al., 2012).

The present study has some limitations. First, our analysis was carried out in the sensor instead of in the source space. Due to the lack of individual structural magnetic resonance images (MRIs), we restricted our analysis to the sensor level. We selected the ROIs for the analysis based on the topographies in the control group, which served as a reference group for the comparison with the dysphoric group. Future studies should investigate potential differences in the sources of brain responses to emotional faces, especially those for M300 between depressed and control participants. In addition, the relatively small sample size warrants a replication of the study with larger participant groups. It is possible that some existing effects were not observable with the current small sample size. It is also worth mentioning that the dysphoric group had depressive symptoms during the measurement, and nearly all of them had a diagnosis of depression. However, the diagnoses were not confirmed in the beginning of the study.

The present study was not designed to determine whether the underlying mechanism related to the vMMN is related to the detection of regularity violations (“genuine vMMN,” Kimura, 2012; Stefanics et al., 2014) or whether it reflects only different levels of neural adaptation in neural populations responding to standard and deviant stimuli (neural adaptation). The most common way to investigate the underlying neural mechanism has been to apply a control condition in which the level of neural adaptation is the same as for the deviant stimulus in the oddball condition, but where no regularity exists (an equiprobable condition, Jacobsen and Schröger, 2001). This control condition has not yet been applied in vMMN studies using facial expressions as a changing feature (some studies have used an equiprobable condition, but the probability of the oddball deviant and control stimulus in the equiprobable condition has been different; Li et al., 2012; Astikainen et al., 2013; Kovarski et al., 2017). Other vMMN studies have used a proper equiprobable condition, and they have demonstrated a genuine vMMN (e.g., for orientation changes, see Astikainen et al., 2008; Kimura et al., 2009). This is an aspect that should be studied in the context of emotional face processing as well. In one study (Kimura et al., 2012), however, the stimulus condition applied allowed neural responses to regularity violations to be observed. Namely, an immediate repetition of an emotional expression was presented as a deviant stimulus violating the pattern of constantly changing (fearful and happy). The vMMN reflecting the detection of the regularity violations was elicited at 280 ms and 350 ms after the stimulus onset for the fearful and the happy faces, respectively. It is thus possible that in our study the differential responses at the two first stages also reflect the neural adaptation to repeatedly presented standard stimuli rather than the genuine vMMN.

In sum, the present results show that there is a negative bias in dysphoria toward rare sad faces, extending the findings of an attentive negative bias in depression to automatic face processing. The results also demonstrate impaired automatic change detection in emotional faces in dysphoria. These findings related to automatic face processing might have significant behavioral relevance that affects, for instance, real-life social interactions.

## Author Contributions

PA and GS conceived and designed the experiments. ER, XL, and KK performed the data acquisition. QX analyzed the data. ER, XL, and CY contributed to the data analysis. PA, QX, CY, KK, GS, and WL interpreted the data. PA and QX drafted the manuscript. All the authors revised and approved the manuscript.

## Conflict of Interest Statement

The authors declare that the research was conducted in the absence of any commercial or financial relationships that could be construed as a potential conflict of interest. The reviewer JK declared a past co-authorship with several of the authors GS, KK, and PA to the handling Editor.
