# Supplementary material for: Automatic Processing of Changes in Facial Emotions in Dysphoria: A Magnetoencephalography Study
Source: Front Hum Neurosci. 2018 May 4;12:186. doi: 10.3389/fnhum.2018.00186 (PMC5945870; doi:10.3389/fnhum.2018.00186)
Supplement: Supplementary file 1 [file Presentation_1.pdf]

![frontiers]

Supplementary Material

Automatic processing of changes in facial emotions in dysphoria: A magnetoencephalography study

Qianru Xu, Elisa M. Ruohonen, Chaoxiong Ye, Xueqiao Li, Kairi Kreegipuu, Gabor Stefanics, Wenbo Luo* & Piia Astikainen

*** Correspondence:** Wenbo Luo: luowb@lnnu.edu.cn

1 Lateralization analysis of the M300

1.1 Statistical analyses

In addition to investigation of the lateralization index with three-way repeated measures ANOVA reported in the main text, the possible lateralization of the M300 responses were further studied with three-way repeated measures ANOVAs separately for happy and sad responses, and deviant and standard responses. For the analyses where happy and sad responses were separately investigated, peak amplitude values of the M300 were applied in the ANOVA model including within-subjects factors Hemisphere (Left vs. Right) and Stimulus type (Standard vs. Deviant) and a between-subject factor Group (Control vs. Dysphoric). For the analyses where standard and deviant responses were separately investigated, peak amplitude values of the M300 were applied in the ANOVA model including within-subjects factors Hemisphere (Left vs. Right) and Emotion (Sad vs. Happy) and a between-subject factor Group (Control vs. Dysphoric).

Furthermore, because small sample size can limit the possibility to observe existing significant differences in multi-way ANOVAs, we also compared lateralization indexes separately for happy (Deviant happy – Standard happy) and sad (Deviant sad – Standard sad) vMMN between the groups with independent samples t-tests (bootstrapping method with 1000 permutations). The lateralization index was calculated with differential responses as follows: Lateralization index = (Left – Right) / (Left + Right).

For all significant ANOVA results, post-hoc analyses were conducted by two-tailed paired t-tests for comparison of the differences involving within-subjects factors and by independent samples t-tests for between-subjects comparisons, and the confidence intervals (CI) were computed with a bootstrapping method using 1000 permutations (Good, 2005).

For all analyses, partial eta-squared (η_p^2) presents effect size estimates for ANOVAs and Cohen's d for t-tests. Cohen's d was computed using pooled standard deviations (Cohen, 1988). In addition, we conducted the Bayes factor analysis to estimate whether the null results in post hoc analyses were observed by chance (Rouder et al., 2009).

1.2 Results

ANOVA for happy and sad face responses

Analysis for happy face responses did not show main effects or interaction effects related to hemisphere (all *p*-values > .234) (see Supplementary Table 1). For sad face responses, an interaction effect of Hemisphere × Stimulus type was significant, $F(1, 19) = 6.36$, $p = .021$, $\eta_p^2 = 0.25$ (see Supplementary Table 1). The main effect of the hemisphere and the other interaction effects with hemisphere were non-significant (all *p*-values > .280). Post hoc analysis for the Hemisphere × Stimulus type interaction effect in sad face responses showed there was only a marginally significant difference, which reflected larger sad deviant responses in the left hemisphere compared to the right hemisphere, $t(20) = 1.77$, $p = .092$, $CI_{95\%}$ [- 53.08, 2.75], $d = 0.44$, $BF_{10} = 0.86$. No other significant results between pairs were found (all *p*-values > .128, all BF_{10}s < 0.67).

ANOVA for deviant and standard responses

Analysis for deviant responses showed neither a main effect of hemisphere nor interaction effects related to it (all *p*-values > .131) (see Supplementary Table 2). Similarly, in standard responses, there was neither a main effect nor interaction effects related to hemisphere (all *p*-values > .471) (Supplementary Table 2).

Lateralization index for the vMMN response

Independent samples t-tests comparing lateralization indexes for the vMMN responses in the dysphoric and control group showed no significant differences between groups in happy, $t(19) = 0.54$, $p = .587$, $CI_{95\%}$ [- 5.30, 3.73], $d = 0.24$, $BF_{10} = 0.32$, or sad vMMN lateralization, $t(19) = 0.865$, $p = .430$, $CI_{95\%}$ [-149.28, 2.50], $d = 0.41$, $BF_{10} = 0.33$ (Supplementary Figure 1). There was one outlier value (more than 4.5 SD) in happy responses in the control group, but the results did not change after removing this participant's value from the analysis, $t(18) = .126$, $p = .901$, $CI_{95\%}$ [-3.96, 3.96], $d = 0.06$, $BF_{10} = 0.40$.

1.3 Summary

In sum, in this supplementary material, we reported the results for the analyses of lateralization of M300 separately for happy and sad, as well as deviant and standard stimulus responses with repeated-measures ANOVAs. In addition, the possible differences in lateralization index for the vMMN was investigated with independent-samples t-tests. There were no significant effects for lateralization in any of the tests. Bayes factor analyses also supported that the null hypotheses are more likely to be true.

References:

Good, P. (2005). *Permutation, Parametric, and Bootstrap Tests of Hypotheses*. 3rd ed. New York: Springer doi:10.1198/jasa.2005.s48.

Rouder, J. N., Speckman, P. L., Sun, D., Morey, R. D., and Iverson, G. (2009). Bayesian t tests for accepting and rejecting the null hypothesis. *Psychon. Bull. Rev.* 16, 225–237. doi:10.3758/PBR.16.2.225.

Supplementary Table 1. ANOVA results for M300 amplitude separately for happy and sad stimulus responses. * $p < .05$

Emotion type	Effect		F (degrees of freedom)	p	η_p^2
Happy Stimuli	Main effects	Hemisphere	F (1,19) = .98	.336	.05
		Stimulus type	F (1,19) = 2.40	.138	.11
		Group	F (1,19) = .72	.407	.04
	Two-way interactions	Hemisphere × Stimulus type	F (1,19) = 1.17	.293	.06
		Hemisphere × Group	F (1,19) = .34	.567	.02
		Stimulus type × Group	F (1,19) = .16	.693	.01
	Three-way interactions	Hemisphere × Stimulus type × Group	F (1,19) = 1.51	.234	.07
Sad Stimuli	Main effects	Hemisphere	F (1,19) = 1.24	.280	.06
		Stimulus type	F (1,19) = .29	.595	.02
		Group	F (1,19) = .18	.677	.01
	Two-way interactions	Hemisphere × Stimulus type	F (1,19) = 6.36	.021*	.25
		Hemisphere × Group	F (1,19) = .02	.900	.001
		Stimulus type × Group	F (1,19) = 3.44	.079	.15
	Three-way interactions	Hemisphere × Stimulus type × Group	F (1,19) = .52	.478	.03

Supplementary Table 2. ANOVA results for M300 amplitude conducted separately for deviant and standard stimulus responses.

Stimulus type	Effect		F (degrees of freedom)	*p*	η_p^2
Deviant Stimuli	Main effects	Hemisphere	F (1,19) = 2.50	.131	.12
		Emotion	F (1,19) = 1.31	.267	.06
		Group	F (1,19) = .10	.760	.01
	Two-way interactions	Hemisphere × Emotion	F (1,19) = 1.15	.298	.06
		Hemisphere × Group	F (1,19) = .40	.537	.02
		Emotion × Group	F (1,19) = 2.74	.114	.13
	Three-way interactions	Hemisphere × Emotion × Group	F (1,19) = 1.18	.291	.06
Standard Stimuli	Main effects	Hemisphere	F (1,19) = .30	.588	.02
		Emotion	F (1,19) = .87	.362	.04
		Group	F (1,19) = .84	.371	.04
	Two-way interactions	Hemisphere × Emotion	F (1,19) = .10	.760	.01
		Hemisphere × Group	F (1,19) = .01	.940	.00
		Emotion × Group	F (1,19) = .78	.389	.04
	Three-way interactions	Hemisphere × Emotion × Group	F (1,19) = .54	.471	.03

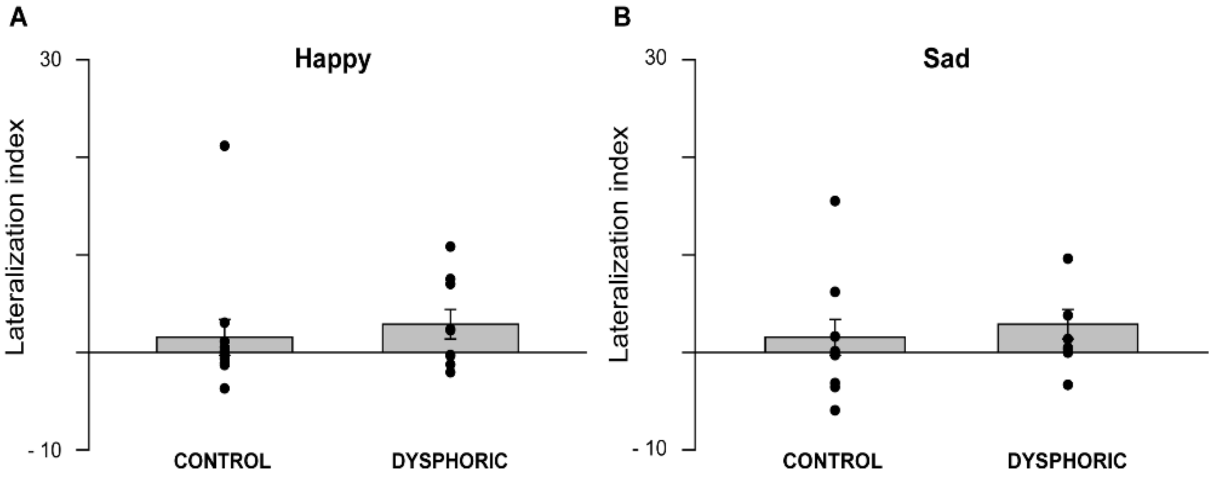

Supplementary Figure 1. Lateralization index (Lateralization index = (Left – Right) / (Left + Right)) for group comparison separately for happy (A) and Sad (B) vMMN responses. The bars present the mean values with standard errors in each group and the dots in the vertical scatter plots represent the lateralization indexes of individual participants. An outlier value of one control participant is removed from figure A. The statistical results do not change significantly if this participant's data is removed from the analysis ($p = .430$ vs. $p =.901$).
